# Opposite effects of a high-fat diet and calorie restriction on ciliary neurotrophic factor signaling in the mouse hypothalamus

**DOI:** 10.3389/fnins.2013.00263

**Published:** 2013-12-27

**Authors:** Ilenia Severi, Jessica Perugini, Eleonora Mondini, Arianna Smorlesi, Andrea Frontini, Saverio Cinti, Antonio Giordano

**Affiliations:** ^1^Department of Experimental and Clinical Medicine, Section of Neuroscience and Cell Biology, Università Politecnica delle MarcheAncona, Italy; ^2^Center of Obesity, Università Politecnica delle Marche-United HospitalsAncona, Italy

**Keywords:** ependyma, tanycytes, third ventricle, median eminence, obesity, fasting, CNTF receptor, STAT3

## Abstract

In the mouse hypothalamus, ciliary neurotrophic factor (CNTF) is mainly expressed by ependymal cells and tanycytes of the ependymal layer covering the third ventricle. Since exogenously administered CNTF causes reduced food intake and weight loss, we tested whether endogenous CNTF might be involved in energy balance regulation. We thus evaluated CNTF production and responsiveness in the hypothalamus of mice fed a high-fat diet (HFD), of *ob*/*ob* obese mice, and of mice fed a calorie restriction (CR) regimen. RT-PCR showed that CNTF mRNA increased significantly in HFD mice and decreased significantly in CR animals. Western blotting confirmed that CNTF expression was higher in HFD mice and reduced in CR mice, but high interindividual variability blunted the significance of these differences. By immunohistochemistry, hypothalamic tuberal and mammillary region tanycytes stained strongly for CNTF in HFD mice, whereas CR mice exhibited markedly reduced staining. RT-PCR and Western blotting disclosed that changes in CNTF expression were paralleled by changes in the expression of its specific receptor, CNTF receptor α (CNTFRα). Injection of recombinant CNTF and detection of phospho-signal transducer and activator of transcription 3 (P-STAT3) showed that CNTF responsiveness by the ependymal layer, mainly by tanycytes, was higher in HFD than CR mice. In addition, in HFD mice CNTF administration induced distinctive STAT3 signaling in a large neuron population located in the dorsomedial and ventromedial nuclei, perifornical area and mammillary body. The hypothalamic expression of CNTF and CNTFRα did not change in the hyperphagic, leptin-deficient *ob*/*ob* obese mice; accordingly, P-STAT3 immunoreactivity in CNTF-treated *ob*/*ob* mice was confined to ependymal layer and arcuate neurons. Collectively, these data suggest that hypothalamic CNTF is involved in controlling the energy balance and that CNTF signaling plays a role in HFD obese mice at specific sites.

## Introduction

Ciliary neurotrophic factor (CNTF) is a 22 kDa cytokine belonging to the interleukin (IL)-6 family, a group of structurally related cytokines that also include IL-11, oncostatin M, leukemia inhibitory factor (LIF), cardiotrophin-1, cardiotrophin-like cytokine and, more recently, neuropoietin (Bauer et al., 2007). CNTF is produced by glial cells in the nervous tissue; its expression is strongest in peripheral nerve Schwann cells (Friedman et al., 1992; Rende et al., 1992) and cerebral white matter astrocytes (Stockli et al., 1991; Guthrie et al., 1997; Dallner et al., 2002). Numerous studies have documented the ability of CNTF to promote cell survival and differentiation in a number of neuronal and glial cell types (Sendtner et al., 1994; Sleeman et al., 2000). Binding of CNTF to a three-part receptor complex (CNTFR) consisting of the ligand-specific binding subunit receptor α (CNTFRα), signal-transducing subunit gp130, and LIF receptor β (Davis et al., 1993; Ip et al., 1993) activates the Janus family of tyrosine kinases (Jak1/Jak2), thereby leading to tyrosine phosphorylation, dimerization and nuclear translocation of signal transducers and activators of transcription (STATs), principally STAT3 (Heinrich et al., 2003; Simi and Ibanez, 2010).

In the peripheral nervous system, CNTF produced by Schwann cells is essential for the postnatal maintenance of motor neurons (Holtmann et al., 2005). Indeed, ablation of the mouse CNTF gene by homologous recombination leads to a progressive atrophy and loss of motor neurons that is more significant in older animals (Masu et al., 1993). In addition, CNTF protects axons in progressive motor neuronopathy mutant mice (Sendtner et al., 1992) and in experimental autoimmune encephalomyelitis (Linker et al., 2002). Therapeutic trials where patients with the motor neuron disease amyotrophic lateral sclerosis were treated with human recombinant CNTF (ACTS, 1996; Miller et al., 1996) failed to provide satisfactory effects on motor performances and were associated with significant side effects, including anorexia and weight loss. Subsequent studies also using Axokine, a modified form of human CNTF with improved potency and stability, confirmed that CNTF administration to humans and experimental animals results in decreased food intake, weight loss, and an improvement of obesity-associated hyperglycaemia, hyperinsulinaemia, and dyslipidaemia (Gloaguen et al., 1997; Lambert et al., 2001; Sleeman et al., 2003; Blüher et al., 2004).

Studies aimed at elucidating the mechanism of action of exogenously administered CNTF or Axokine stressed the role(s) of CNTF in the hypothalamus, the key region for energy balance regulation and homeostasis. It was suggested that the anorectic response to CNTF could be due to a leptin-like action in the hypothalamus via activation of Jak1/Jak2-STAT3 signaling in arcuate nucleus neurons (Lambert et al., 2001; Janoschek et al., 2006) and/or to production of novel neurons capable of integration in the leptin-dependent neuronal circuits regulating the energy balance (Kokoeva et al., 2005). Recently however, CNTFRα deletion in hypothalamic leptin receptor-expressing neurons failed to impair the anorectic effect of Axokine (Stefater et al., 2012). Thus, CNTF may also act on hypothalamic targets other than those affected by leptin.

In a recent paper (Severi et al., 2012), we showed that CNTF is constitutively expressed by glial cells in the mouse hypothalamus. In normal mice CNTF was detected by immunohistochemistry in the ependymal layer throughout the rostro-caudal extension of the third ventricle in numerous tanycytes and multiciliated ependymal cells. Some astrocytes, mainly located in a subependymal position, were also CNTF-positive. In addition, treatment with recombinant CNTF and detection of phospho-STAT3 (P-STAT3) immunoreactivity showed that multiciliated ependymal cells and tanycytes producing CNTF are also CNTF-responsive. A distinctive pattern was seen in the tuberal region of the mouse hypothalamus, where numerous CNTF-producing cells were adjacent to and overlapped to a considerable extent with CNTF-responsive ependymal cells. To assess whether endogenously-produced CNTF has a role in the pathophysiological control of the energy balance, here we set out to evaluate whether hypothalamic CNTF signaling is affected by different energy balance challenges. In particular, we examined the expression of CNTF and CNTFRα, and the distribution of CNTF-producing and CNTF-responsive cells in the hypothalamus of mice rendered obese by a high-fat diet (HFD) or by leptin-deficiency (*ob*/*ob* mice), and in mice fed a calorie restriction (CR) diet. Collectively, our findings suggest that HFD obesity is associated with an increase in hypothalamic CNTF signaling, whereas CR induces a reduction of CNTF signaling in the hypothalamus. In contrast to HFD, the hyperphagic obesity of *ob*/*ob* mice did not appear to be associated with modifications in CNTF signaling.

## Materials and methods

### Animals and experimental conditions

Four-week-old male Swiss CD-1 mice purchased from Charles River (Calco, Italy) were housed individually in plastic cages under constant environmental conditions (12 h light/dark cycle at 22°C) with *ad libitum* access to food and water. Handling was limited to cage cleaning. After one week of acclimatization, animals were weighed and divided into three groups with approximately equal mean body weight: one group was fed an HFD (Charles River; 50 kJ% from fat, 30 kJ% from carbohydrates and 20 kJ% from proteins; HFD mice); another group was fed a low-fat diet (Charles River; 19 kJ% from fat, 50 kJ% from carbohydrates and 31 kJ% from proteins; control mice), and the third group was fed about 60% of the normal daily amount of the low-fat food normalized for weight and age (CR mice). CR mice were fed from 7.00 to 8.00 am, whereas HFD and control mice had *ad libitum* access to their food. These feeding conditions were maintained for 12 weeks. Male C57BL/6 wild type, C57BL/6-Lep^°b^ *ob*/+ and C57BL/6-Lep^°b^ *ob*/*ob* mice purchased from Charles River at 4 weeks of age were housed individually and kept under constant environmental conditions with free access to standard chow diet and water. They were used for experimental procedures at 12–14 weeks of age. All animals were sacrificed in a fed state between 11.00 and 12.00 am. All efforts were made to minimize animal suffering and to reduce the number of animals used. Experiments were carried out in accordance with EC Council Directive 86/609/EEC of 24 November 1986.

For RT-PCR and Western blot assays animals were decapitated, the brain was rapidly removed from the skull, placed with its ventral side up in a pre-cooled adult mouse coronal brain matrix (ASI Instruments, Warren, MI, USA) and the hypothalamus was dissected out. Histological examination confirmed that these specimens consisted of all parts of the hypothalamus—from the preoptic area to the mammillary body—including the whole third ventricle. The remaining part of the brain was also collected as a whole. Specimens were immediately snap-frozen in liquid nitrogen and stored at −80^°^C. For morphological studies, animals were anaesthetized with 100 mg/kg ketamine (Ketavet, Farm. Gellini, Aprilia, Italy) in combination with 10 mg/kg xylazine (Rompum, Bayer AG, Leverkusen, Germany) and perfused transcardially with 4% paraformaldehyde in 0.1 M phosphate buffer (PB), pH 7.4. Brains were carefully removed from the skull, postfixed with the same fixative solution for 24 h at 4°C and washed in PB. Free-floating coronal sections (40-μm-thick) of the entire brain from the septum to the mammillary body of the hypothalamus were obtained with a Leica VT1200S vibratome (Leica Microsystems, Vienna, Austria) and kept in phosphate buffered saline (PBS), pH 7.4, at 4°C until use.

Before sacrifice, some mice received a single intraperitoneal injection (0.3 mg/kg of body weight) of recombinant rat CNTF (R&D Systems, Minneapolis, MN, USA) or pyrogen-free saline using a Hamilton syringe. The volumes of CNTF, or vehicle, ranged from 180 to 360 μl according to body weight. Forty-five minutes later mice were anaesthetized and processed for morphological analyses as described above.

### Reverse transcription-polymerase chain reaction (RT-PCR)

Total RNA was extracted from samples after homogenization using TRIZOL reagent (Invitrogen, Milano, Italy), purified, digested with ribonuclease-free deoxyribonuclease and concentrated using RNeasy Micro kit (Qiagen, Milano, Italy) according to the respective manufacturer's instructions.

For determination of mRNA levels, 1 μg of RNA was reverse-transcribed with a High-Capacity cDNA RT Kit with RNase Inhibitor (Applied BioSystems, Foster City, CA, USA) in a total volume of 20 μl. Real time gene expression was analyzed in triplicate by using TaqMan Gene Expression Assays (Applied BioSystems) as listed: TATA box binding protein (TBP): Mm00446973_m1; CNTF: Mm00446373_m1; CNTFRα: Mm00516693_m1; neuropeptide Y (NPY): Mm03048253_m1; proopiomelanocortin (POMC): Mm00435874_m1 and Master Mix TaqMan (Applied BioSystems).

Reactions were carried out in an ABI 7300 system (Applied Biosystems) using 50 ng of RNA in a final reaction volume of 20 μl and the following thermal cycle protocol: initial incubation at 95°C −10 min, followed by 40 cycles of 95°C −15 s and 60°C −20 s. In order to rule out genomic contamination, a control reaction where reverse transcriptase was omitted in the amplification mixture was included for each sample. Relative mRNA expression was determined by the ΔΔ-Ct method using TBP levels as an endogenous control. Differences in starting total RNA and in cDNA synthesis efficiency among samples were normalized using TBP expression. Results were expressed as fold changes in relative gene expression compared with the control group. Data are presented as histograms ± standard error of the mean (SEM).

### Primary antibodies

The following primary antibodies were used in the study: anti-CNTF polyclonal goat serum (R&D Systems, cat # AF-557-NA); anti-CNTF polyclonal rabbit serum (cat # ab46172); anti-CNTFRα polyclonal rabbit serum (both from Abcam, Cambridge, UK, cat # ab127425); anti-glial fibrillary acidic protein (GFAP) monoclonal mouse antibody (Sigma, St Louis, MO, USA, cat # G3893); anti-phospho-specific-(Tyr705)-STAT3 polyclonal rabbit serum (Cell Signaling Technology Inc., Beverly, MA, USA, cat # 9131); anti-β-tubulin monoclonal mouse antibody (Pierce, Rockford, IL,USA, cat # MA5-16308); anti-HuC/D monoclonal mouse antibody (Molecular Probes, Eugene, OR, USA, cat # A21271).

### Western blot analysis

Proteins were isolated from the phenol-ethanol supernatant obtained using TRIZOL reagent according to the manufacturer's instructions. Soluble protein was quantified using a Bradford protein assay (Bio-Rad, Richmond, CA, USA) and equal amounts of proteins were loaded onto 12-well Bolt® 4–12% Bis-Tris Plus Gels (Invitrogen) and resolved by SDS–PAGE. Proteins were transferred to 0.2 mm nitrocellulose membranes using iBlot® System EU (Invitrogen) according to the manufacturer's instructions. After blocking the membrane with 5% non-fat dry milk (Bio-Rad) in Tris-buffered saline with 0.1% Tween 20 (TBS-T), membranes were incubated overnight at 4°C with the primary antibodies: polyclonal rabbit anti-CNTF (diluted 1:1500), polyclonal rabbit anti-CNTFRα (diluted 1:500) or monoclonal mouse anti-β-tubulin (diluted 1:3000). Membranes were extensively washed with TBS-T 0.1% and then incubated for 2 h with the secondary antibodies: horseradish peroxidase-conjugated goat anti-rabbit IgG (Vector Laboratories, Burlingame, CA, USA; diluted 1:1000; CNTF and CNTFRα schedules) or horseradish peroxidase-conjugated goat anti-mouse IgG (Bethyl Laboratories, Montgomery, TX, USA; diluted 1:20,000; β-tubulin schedule). Specific immunoreactivity was visualized using Super Signal West Pico chemiluminescent substrate (Pierce). Protein levels were assessed in at least three separate experiments per molecule by densitometric analysis using Chemidoc and Quantity-One program (Bio-Rad). Quantities were normalized to β-tubulin expression. Results were expressed as fold changes in relative protein expression compared with the control group. Data are shown as histograms ± SEM.

### Peroxidase immunohistochemistry

Immunohistochemical detection of CNTF was performed according to standard procedures. In brief, free-floating sections were reacted with 0.3% H_2_O_2_ (in PBS; 30 min) to block endogenous peroxidase, rinsed with PBS and incubated with 3% normal serum blocking solution (in PBS; 60 min). Then they were incubated with the anti-CNTF polyclonal goat serum (dilution 1:100) in PBS, overnight at 4°C. After a thorough rinse in PBS, sections were incubated in 1:200 v/v biotinylated secondary antibody solution (in PBS; 30 min), rinsed in PBS and incubated in avidin-biotin peroxidase complex (ABC Elite PK6100, Vector), washed several times in PBS and finally incubated in 3,3′ diaminobenzidine tetrahydrochloride (0.05% in 0.05 M Tris with 0.03% H_2_O_2_; 5 min). After immunohistochemical staining, sections were mounted on slides, air-dried, dehydrated in ethanol, cleared with xylene and covered with Entellan. The ability of this antibody specifically to detect CNTF in mouse brain has previously been shown by our group (Severi et al., 2012).

Unmasking procedures were used for P-STAT3 immunohistochemical detection (Frontini et al., 2008). Free-floating sections were reacted with 1% NaOH and 1% H_2_O_2_ (20 min), 0.3% glycine (10 min) and 0.03% sodium dodecyl sulfate (10 min). After rinsing in PBS, they were blocked with 3% normal goat serum (in 0.2% Triton X-100; 60 min) and incubated with polyclonal anti-P-STAT3 rabbit serum (dilution 1:1000) in PBS, overnight at 4°C. The next day, the procedure was performed as described above. Staining was not observed when the primary antibody was omitted.

### Immunofluorescence and confocal microscopy

For double-labeling experiments, free-floating sections were processed according to the P-STAT3 protocol until the incubation with the primary antibody. Then they were incubated overnight in a mixture of two primary antibodies: polyclonal goat anti-CNTF (1:50) and monoclonal mouse anti-GFAP antibody (1:1000); polyclonal goat anti-CNTF (1:50) and polyclonal rabbit anti-P-STAT3 (1:700) or polyclonal rabbit anti-P-STAT3 (1:700) and mouse monoclonal anti HuC/D (1:100). The next day sections were washed twice with PBS and incubated in a cocktail of fluorophore-linked secondary antibodies at a dilution of 1:100 in PBS for 1 h at room temperature. The secondary antibodies were DyLight™488-conjugated anti-goat IgG, DyLight™549-conjugated anti-mouse IgG, DyLight™488-conjugated anti-rabbit IgG and DyLight™649-conjugated anti-rabbit IgG (all from Jackson ImmunoResearch, West Grove, PA, USA). Sections were subsequently washed twice with PBS, mounted on standard glass slides, air-dried and coverslipped using Vectashield mounting medium (Vector). Sections were viewed under a motorized Leica DM6000 microscope at different magnifications. Fluorescence was detected with a Leica TCS-SL spectral confocal microscope (Leica Microsystems) equipped with an Argon and He/Ne mixed gas laser. Fluorophores were excited with the 488, 543, and 649 nm lines and imaged separately. Images (1024 × 1024 pixels) were obtained sequentially from two channels using a confocal pinhole of 1.1200 and stored as TIFF files. Brightness and contrast of the final images were adjusted using Photoshop 6 (Adobe Systems, Mountain View, CA, USA). The percentages of GFAP-positive tanycytes and astrocytes also expressing CNTF and the percentage of GFAP-positive tanycytes also expressing P-STAT3 were calculated in 5 alternate double-stained coronal sections of the tuberal portion of the hypothalamus from 5 mice per experimental group.

### Statistical analysis

Differences between groups were evaluated by One-Way ANOVA. Statistical analysis was performed with GraphPad Prism (version 5.00 for Windows) software (San Diego, CA, USA). A level of *p* < 0.05 was considered significant.

## Results

### CNTF-producing cells in the hypothalamus of HFD mice

**Figure 1:**
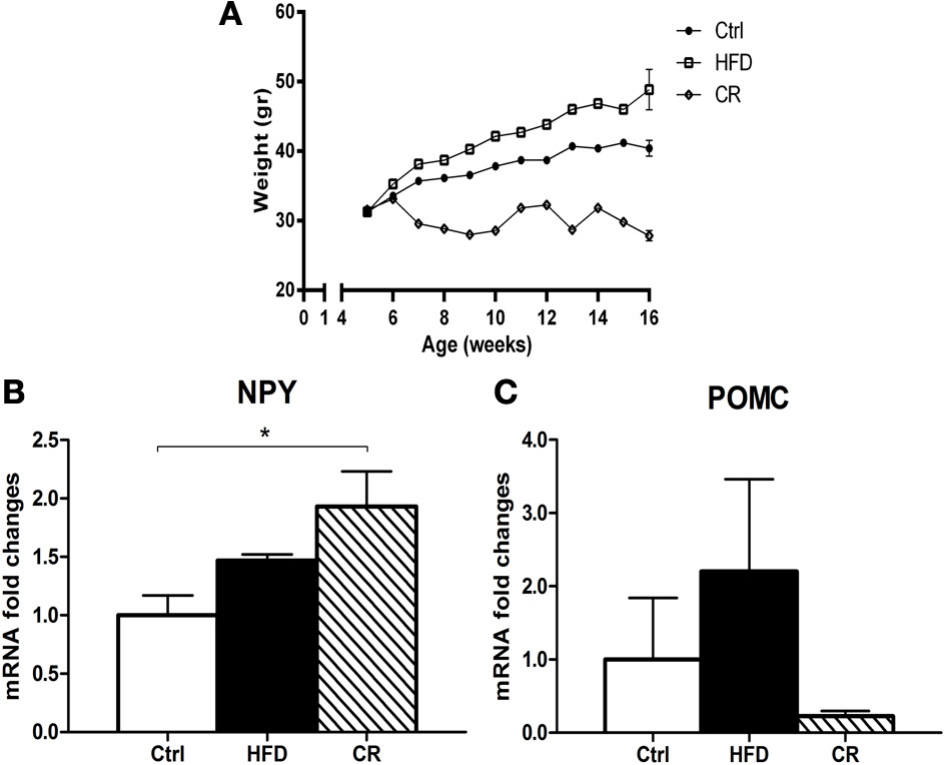
**(A) trends of the body weight of mice fed an HFD diet, a low-fat diet (Ctrl), or a CR regimen for 12 weeks.** RT-PCR analysis of NPY **(B)** and POMC **(C)** expression in the hypothalamus of HFD, control (Ctrl) and CR mice. Mean ± SEM. ^*^P < 0.05.

**Figure 2:**
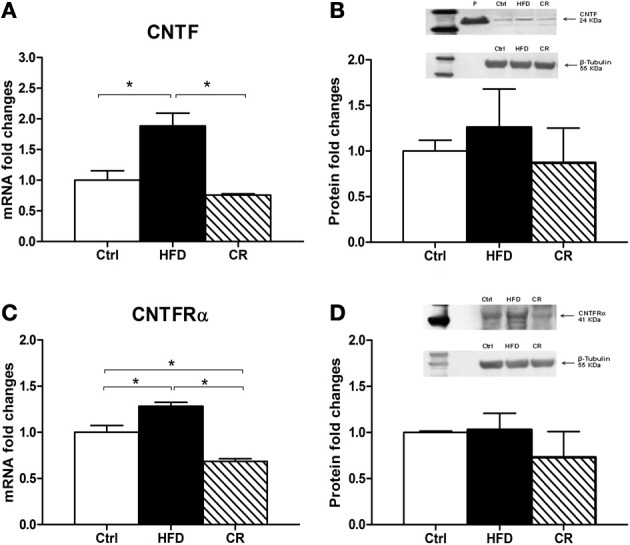
**RT-PCR (A and C) and Western blot (B and D) analyses of CNTF (A and B) and CNTFRα (C and D) expression in the hypothalamus of HFD, control (Ctrl) and CR mice.** For Western blots the densitometric analysis was normalized to β-tubulin expression. In **(B)** recombinant CNTF (P, 30 ng) loaded together with hypothalamic protein extracts served as a positive control. Mean ± SEM. ^*^P < 0.05.

After 12 weeks on the HFD mice weighed significantly more than controls (Figure 1A). The hypothalamic expression of NPY mRNA (Figure 1B) and POMC mRNA (Figure 1C), the latter coding for the anorexigenic peptide α-MSH, was higher in HFD mice than in control animals, but the increase was not statistically significant. CNTF mRNA was significantly increased in the hypothalamus of HFD mice and was almost double that seen in controls (Figure 2A). The increase appeared to be restricted to the hypothalamus, because no differences in CNTF expression were found in the samples containing the remaining brain portions (data not shown). Western blot analysis of hypothalamic protein extracts confirmed that CNTF expression was higher in HFD than in control mouse hypothalamus, although the difference was not significant due to a high interindividual variability among HFD mice (Figure 2B).

**Figure 3:**
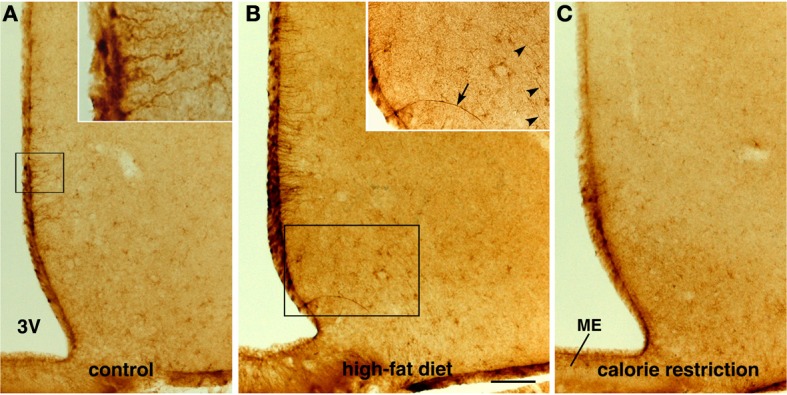
**Immunohistochemical detection of CNTF in the mouse tuberal hypothalamus.** Some CNTF-stained tanycytes on the ventrolateral wall of the third ventricle (3V) in control mouse **(A)**. In an HFD mouse **(B)**, strongly increased CNTF staining of tanycytes also results in more (inset of **B**, arrow) or less (inset of **B**, arrowheads) intense staining of their long projections. In contrast, tanycytes in CR mice **(C)** exhibit very low-level staining. The insets of **(A,B)** are the enlargements of the corresponding framed areas. ME, median eminence. Bar: **(A–C)** 400 μm; inset of **(A)** 40 μm; inset of **(B)** 300 μm.

**Figure 4:**
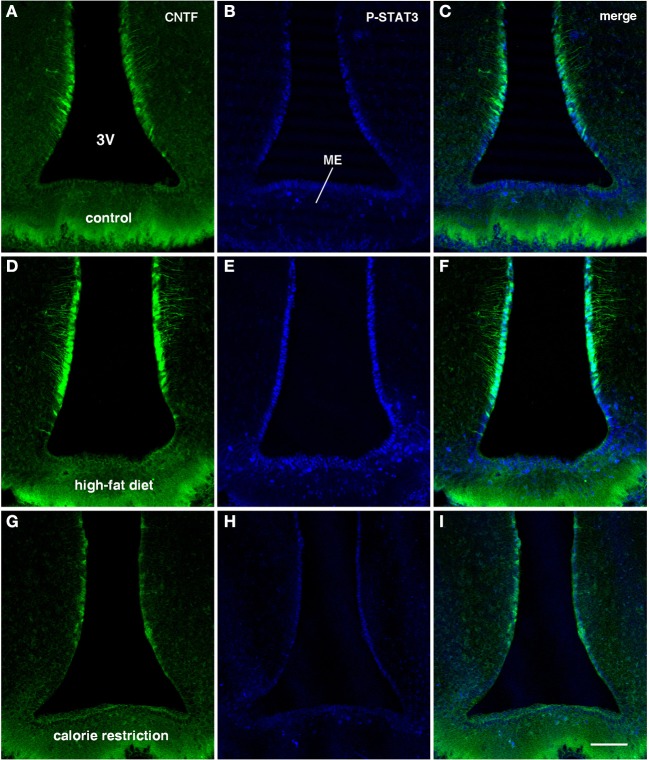
**CNTF/P-STAT3 double-labeling of coronal sections of the tuberal hypothalamus from CNTF-treated control (A–C), HFD (D–F) and CR (G–I) mice.** Compared with controls **(A,B)** the greater CNTF expression seen in tanycytes of HFD mice **(D)** is matched by increased CNTF responsiveness **(E)**. In CR mice both CNTF expression **(G)** and responsiveness **(H)** are lower than in control mice and, especially, HFD mice. Bar: 100 μm.

**Figure 5:**
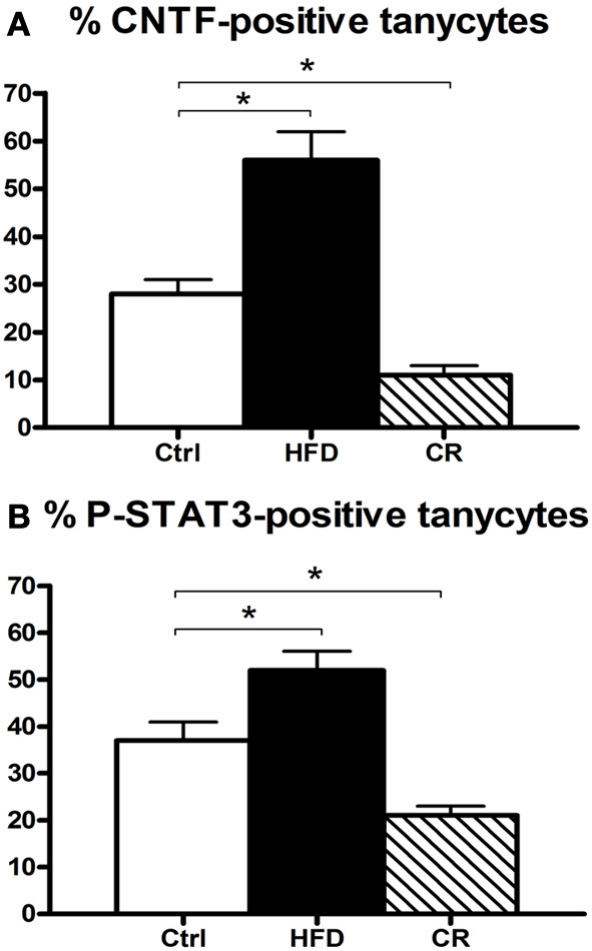
**Percentage of CNTF-producing (A) and CNTF-responsive (B) tuberal and mammillary tanycytes in HFD, control (Ctrl) and CR mice.** Morphometric evaluation performed by confocal microscopy in double-stained sections where tanycytes were labeled by GFAP and CNTF responsiveness was evaluated by P-STAT3 immunohistochemistry in CNTF-treated mice. Mean ± SEM. ^*^P < 0.05.

CNTF was detected by immunohistochemistry in the ependymal cells and tanycytes of the third ventricle wall and in subependymal astrocytes in the hypothalamus of both control (Severi et al., 2012) and HFD mice. Comparison of corresponding coronal brain sections, processed in the same immunohistochemical experiment under standardized conditions, disclosed that while similar levels of CNTF staining were found in the preoptic and anterior hypothalamus of HFD and control mice, CNTF staining was stronger in the tuberal and mammillary regions of HFD mice. In particular, intensely stained tanycytes lining the ventrolateral walls of the third ventricle were detected in the ependymal layer of the tuberal region of the hypothalamus of HFD mice (compare Figure 3B with Figure 3A and Figure 4D with Figure 4A); sometimes their long projections into the underlying gray matter were also variably stained (inset of Figure 3B). Additionally, astrocytes located in the arcuate nucleus were more intensely stained in HFD than in control mice. Double-staining experiments and morphometry disclosed that the percentage of GFAP-positive tanycytes that were also positive for CNTF increased significantly in HFD mice (Figure 5A), whereas the number of astrocytes also expressing CNTF did not change (data not shown). Collectively, these data document that HFD induces an increased CNTF expression that is especially evident in the tanycytes of the tuberal and mammillary regions.

### CNTF-responsive cells in the hypothalamus of HFD mice

In order to gain further insights into the role played by CNTF in the hypothalamus of mice fed an HFD we evaluated the expression of its specific receptor, CNTFRα. A significant increase of CNTFRα mRNA was found in the hypothalamus of HFD mice compared with controls (Figure 2C), whereas no differences in CNTFRα mRNA levels were detected in the extracts of the remaining brain portions (data not shown). In contrast to RT-PCR data, Western blotting showed no difference in CNTFRα protein content between HFD and control mice (Figure 2D).

**Figure 6:**
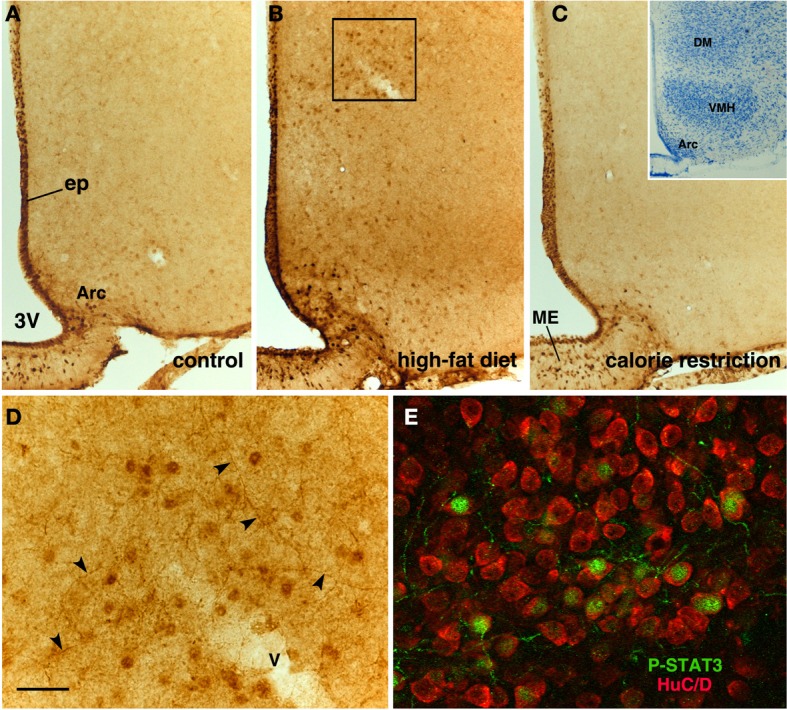
**P-STAT3 immunohistochemistry in the tuberal region of CNTF-treated mice.** In a control mouse **(A)**, P-STAT3 staining depicts ependymal cells (ep) and neurons located in the arcuate nucleus (Arc) and median eminence (ME). In an HFD mouse **(B)**, P-STAT3 immunoreactivity increases at these sites, but responsiveness to CNTF is also detectable in the dorsomedial hypothalamus (DM) and, to a lesser extent, the ventromedial nucleus (VMH). CNTF responsiveness is blunted in a CR mouse **(C)**. The inset of **(C)** shows a representative Nissl-stained coronal section of the tuberal region of the hypothalamus. **(D)** is the enlargement of the area framed in **(B)**, where P-STAT3 immunoreactivity is found not only in cell nuclei but also in varicose projections (arrowheads). In **(E)**, double-staining immunofluorescence and confocal microscopy show that in the DM of a CNTF-treated HFD mouse P-STAT3-positive cell nuclei are contained in HuC/D-positive neuronal cell bodies. 3V, third ventricle; v, blood vessel. Bar: **(A–C)** 100 μm; **(D)** 20 μm; **(E)** 15 μm; inset of **(C)** 400 μm.

Intraperitoneally or intravenously injected CNTF rapidly reaches distinctive areas of the brain parenchyma by crossing the blood-cerebrospinal fluid barrier and the blood-brain barrier (Poduslo and Curran, 1996; Pan et al., 1999; Lambert et al., 2001). As a consequence, detection of nuclear P-STAT3 immunoreactivity after systemic CNTF treatment is a reliable neuroanatomical tool to obtain a functional mapping of the central actions of CNTF and to characterize CNTF-responsive, CNTFRα-bearing cells (MacLennan et al., 2000). Similar to control mice (Severi et al., 2012), P-STAT3 immunoreactivity was detected in arcuate nucleus neurons, ependymal cells and tanycytes of CNTF-treated HFD mice. Very few astrocytes were activated. Ependymal STAT3 signaling following CNTF injection was stronger in the tuberal and mammillary regions of HFD than in control mice (compare Figure 4E with Figure 4B, Figure 4F with Figure 4C and Figure 6B with Figure 6A). Indeed, the percentage of tanycytes also responding to exogenously administered CNTF was significantly higher in HFD than control mice (Figure 5B). Most importantly, CNTF administration to HFD obese mice induced STAT3 activation in a large population of adjacent cells in the gray matter of the tuberal and mammillary regions of the hypothalamus (Figure 6B). These CNTF-responding cells, showing specific nuclear P-STAT3 staining, were characterized by round nuclei with a frequently unstained nucleolus whose profile resembled that of neurons (Figure 6D). P-STAT3-positive cell projections were also noted among positive cells (Figure 6D, arrowheads). All these cells were also positive for the neuronal marker HuC/D (Figure 6E), which confirmed that they were indeed neurons. Confocal microscopy examination also allowed identification of P-STAT3-positive projections as neuronal projections. Nissl staining of adjacent brain sections demonstrated that CNTF-responsive neurons were located in the ventromedial (VMH) and dorsomedial (DM) nuclei, perifornical area and gray matter of the mammillary body. Consequently, the higher CNTF expression seen in the ependyma of the tuberal and mammillary regions of the HFD mouse hypothalamus is matched by an increased CNTF responsiveness. In particular, exogenously administered CNTF elicited a response from HFD mouse neurons that were never activated in control mice. On the whole, these data suggest that CNTF signaling increases in the hypothalamus of HFD mice.

### CNTF signaling in the hypothalamus of CR mice

If an HFD induces increased hypothalamic CNTF signaling, CR is expected to exert the opposite effect. After 12 weeks, the body weight of CR mice was significantly lower than that of control mice (Figure 1A). Similar to fasted mice (Mizuno et al., 1999), CR mice exhibited significantly higher mRNA levels of the orexigenic peptide NPY (Figure 1B) and non-significantly lower levels of the anorexigenic POMC peptide compared with control animals (Figure 1C). Hypothalamic CNTF mRNA levels were lower in CR mice than in control mice and HFD mice, but the difference was significant only in the latter animals (Figure 2A). In addition, CNTFRα mRNA decreased significantly in CR compared with control and HFD mice (Figure 2C), whereas CNTF and CNTFRα levels showed no differences in samples containing the remaining parts of the brain (data not shown). Western blot analysis showed a reduction of both CNTF (Figure 2B) and CNTFRα (Figure 2D) in the hypothalamus of CR mice, but here too the high SEM involved that the difference was not significant.

As in controls, CNTF was detected by immunohistochemistry in hypothalamic ependymal cells, tanycytes and astrocytes. Comparison of corresponding sections revealed that ependymal CNTF staining in the tuberal and mammillary parts of the hypothalamus was lower in CR than in control mice (compare Figure 3C with Figure 3A and Figure 4G with Figure 4A). Accordingly, the number of tanycytes also expressing CNTF showed a significant reduction (Figure 5A). CNTF administration induced a weaker activation of STAT3 signaling in the ependymal wall and arcuate nucleus (compare Figure 4H with Figure 4B, Figure 4I with Figure 4C and Figure 6C with Figure 6A) and the number of P-STAT3-positive tanycytes significantly reduced (Figure 5B). P-STAT3-positive neurons were never observed outside the arcuate nucleus in these mice.

### CNTF signaling in the hypothalamus of ob/ob mice

**Figure 7:**
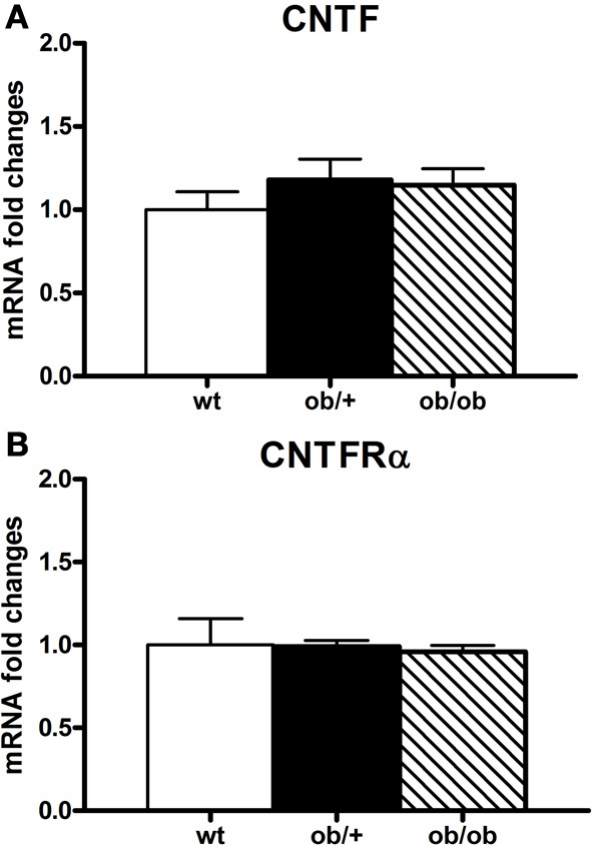
**RT-PCR analysis of CNTF (A) and CNTFRα (B) in the hypothalamus of wild type (wt), *ob*/+ and *ob*/*ob* mice.** Mean ± SEM.

Besides HFD mice, which are the experimental model most similar to human obesity, another widely employed experimental model of obesity is *ob*/*ob* mice, where genetic leptin deficiency induces severe obesity as well as a number of other metabolic and endocrine abnormalities, such as hypothyroidism and hypogonadism. Examination of the indices of CNTF signaling disclosed the absence of any changes in CNTF or CNTFRα mRNA (Figure 7) or protein expression in the hypothalamus of *ob*/*ob* vs. wild type lean mice. By immunohistochemistry, CNTF was expressed by hypothalamic ependyma (including tanycytes) and astrocytes, without differences between *ob*/*ob* and wild type mice (data not shown). Finally, CNTF administration in these mice activated ependymal cells, tanycytes and arcuate nucleus neurons, but never the neuron populations that were found to be CNTF-responsive in HFD obese mice.

## Discussion

The widespread occurrence of obesity and associated diseases requires urgent investigation of the central mechanisms involved in energy balance regulation. The tuberal region of the hypothalamus, containing the arcuate nucleus, VMH, DM and the lateral hypothalamic area, is a prominent brain region for the integration of metabolically relevant stimuli (Williams and Elmquist, 2012). The arcuate nucleus, lying adjacent to the bottom of the third ventricle and to the median eminence, is a circumventricular organ lacking the blood-brain barrier that is ideally located to sense metabolically relevant molecules in the circulation. It contains peptidergic neurons that respond to acute and long-term metabolic inputs in opposite ways: NPY neurons strongly stimulate food intake whereas POMC neurons, by cleaving the POMC gene product into α-MSH, induce a strong reduction in food intake and increase energy expenditure. Arcuate nucleus neurons massively project to the paraventricular nucleus, VMH, DM and the lateral hypothalamic area, where additional second-order peptidergic neuronal systems participate in energy balance control (Schwartz et al., 2000; Sohn et al., 2013).

We previously showed that CNTF is expressed in the ependyma of the third ventricle and in astrocytes of the normal mouse hypothalamus (Severi et al., 2012). Here we show that CNTF expression significantly increases in the ependymal layer of the tuberal and mammillary regions of mice rendered obese by an HFD and that it significantly decreases in mice kept in CR conditions. Interestingly, changes in CNTF expression were paralleled by changes in its specific receptor, CNTFRα; as a result hypothalamic responsiveness to CNTF was greater in HFD than in CR mice. Collectively, these data suggest that in mice an HFD is associated with increased CNTF signaling in the hypothalamus, whereas CR is associated with reduced hypothalamic CNTF signaling. Even though the metabolically relevant stimuli (nutrients and/or hormones) that are capable to induce such changes in CNTF signaling are still unclear, the present findings support the notion that CNTF is a novel, glial-derived modulator of the energy balance. By acting at the central level, administration of CNTF or Axokine reduces food intake (Gloaguen et al., 1997; Lambert et al., 2001; Sleeman et al., 2003; Blüher et al., 2004) and increases energy expenditure (Solymár et al., 2011). Thus, upregulation of CNTF signaling in the hypothalamus of HFD mice may be viewed as a compensatory mechanism counteracting the positive energy balance.

In this study we used HFD mice because they are considered as the most representative model of human obesity. Indeed, the modern human diet is rich in saturated fat, especially in Western countries, and mice fed an HFD for a prolonged period develop an obesity syndrome showing all the hallmarks of human obesity, such as leptin resistance, impaired glucose tolerance, dyslipidaemia, hypertension, hepatic steatosis and adipose tissue inflammation (Panchal and Brown, 2011; Giordano et al., 2013). In contrast, *ob*/*ob* mice show severe hyperphagic obesity due to the genetic absence of the satiety factor leptin. Interestingly, unlike their HFD obese counterparts these mice showed no evidence of increased hypothalamic CNTF signaling. This suggests that leptin does not affect hypothalamic CNTF signaling and also that diet composition may affect CNTF expression and signaling to a greater degree than food amount in the mouse hypothalamus.

The lack of change in the expression of CNTF and CNTFRα seen in *ob/ob* mice is also in line with the ability of CNTF to reduce food intake in these animals (Lambert et al., 2001). On the other hand, CNTFRα deletion in hypothalamic leptin receptor-expressing neurons does not impair the anorectic effect of Axokine (Stefater et al., 2012). Collectively, these data provide further support for the fact that the hypothalamic effects of CNTF on the energy balance are independent of leptin signaling. Thus, a greater knowledge of CNTF-mediated regulation of the energy balance may suggest new therapeutic strategies for human leptin-resistant obesity.

That endogenous CNTF could have a role as a modulator of the energy balance in the hypothalamus was first surmised by Vacher et al. (2008), who also found CNTF upregulation in the hypothalamus of rats rendered obese by a high-sucrose diet and ascribed it to neurons and astrocytes in the arcuate nucleus (Vacher et al., 2008). Even though we detected CNTF immunoreactivity in mouse arcuate nucleus astrocytes, we found no CNTF-positive neurons in this nucleus or in any other region of the mouse hypothalamus. Thus, CNTF production by hypothalamic neurons remains to be demonstrated. However, our immunohistochemical data agree with findings obtained in other CNS regions, where CNTF is a glial-derived neurotrophic factor, at least in normal conditions (Sleeman et al., 2000).

Indeed, *in situ* hybridization and immunohistochemistry have documented that mouse CNTF expression is confined to glial cells in the brain, including astrocytes and ependymal layer cells (Guthrie et al., 1997; Dallner et al., 2002; Severi et al., 2012). We demonstrated CNTF upregulation in HFD mice, mainly in tanycytes of the tuberal and mammillary regions of the hypothalamus. In addition, tanycytes and ependymal cells at these sites were also more responsive to CNTF, as reflected by the stronger P-STAT3 expression found in CNTF-treated obese mice. The close spatial relationship of CNTF-producing and CNTF-responding cells suggests that a local, CNTF-dependent, autocrine-paracrine circuit may be recruited in situations of positive energy balance. The opposite mechanism is likely to be activated in conditions of chronic negative energy balance (CR mice).

Hypothalamic tanycytes are a heterogeneous population of glial cells that constitute the main cell type in the ependymal layer of the tuberal and mammillary regions of the hypothalamus. They are characterized by a cell body located in the ependymal layer and by a single process branching into the underlying neuropil. Recently it has been suggested that third ventricle tanycytes, by affecting neurogenesis, may have a role in the neural control of the energy balance (Bolborea and Dale, 2013). Neurogenesis occurs in the adult hypothalamus and contributes to the plasticity and remodeling of the hypothalamic neuronal networks involved in the control of feeding and energy expenditure (Kokoeva et al., 2005; Xu et al., 2005; Perez-Martin et al., 2010; Pierce and Xu, 2010; Lee et al., 2012). Although the identity and location of adult hypothalamic stem/progenitor cells is yet to be clarified, recent studies suggest that neurogenic niche-like structures may be found along the third ventricle wall (Xu et al., 2005; Migaud et al., 2010; Lee et al., 2012), where tanycytes are good candidates for the role of stem/progenitor cells. In particular, tanycytes are remnants of the radial glial cells that during development serve as neural progenitors and form a scaffold for the migration of newly generated neurons (Kriegstein and Alvarez-Buylla, 2009). In the mature brain they retain the expression of neural stem cell markers such as nestin, vimentin and doublecortin-like proteins (Rodríguez et al., 2005; Saaltink et al., 2012). They also express Notch pathway components and hypothalamic progenitor-specific transcription factors (Shimogori et al., 2010). Fate mapping experiments using BrdU showed that tanycytes can proliferate in response to basic fibroblast growth factor (b-FGF; Xu et al., 2005) and insulin-like growth factor I (Perez-Martin et al., 2010). Finally, genetic fate mapping studies showed that a subset of median eminence tanycytes can generate neurons in newborns (Lee et al., 2012); in addition, the proliferation properties of a subpopulation of α-tanycytes has been shown to depend on FGF signaling (Robins et al., 2013). Our findings, showing that tanycytes are responsive to CNTF, suggest that the enhanced hypothalamic neurogenesis linked to intracerebroventricular infusion of CNTF (Kokoeva et al., 2005) relies on the action of exogenous CNTF on tuberal and mammillary region tanycytes. On the other hand, our data show that tanycytes do not only respond to CNTF but also produce it, especially in the HFD condition. Notably, upregulation of median eminence neurogenesis has been shown to occur in response to HFD in mice (Lee et al., 2012). Thus, the increased CNTF signaling detected in tanycytes located close to the median eminence in our HFD obese mice lends support to the hypothesis that CNTF signaling is a molecular pathway recruited by the HFD to enhance neurogenesis in the hypothalamus.

A distinctive finding of our study was that a large population of neurons located outside the arcuate nucleus, specifically in DM, VMH, the perifornical area and mammillary body, develop responsiveness to CNTF in HFD mice. Importantly, when hypothalamic CNTF signaling did not change, as in *ob*/*ob* obese mice, or decreased, as in CR mice, these neurons did not respond to CNTF. Further investigation is required to characterize the phenotype of these neurons and find whether CNTF activates or inhibits them. However our data highlight novel and specific targets of CNTF, or Axokine, in the brain of HFD mice. An interesting question arising from these findings is the putative endogenous source of CNTF that can affect this neuronal population in the HFD condition. The present data point to tanycytes as the major CNTF-producing cell type in the tuberal and mammillary hypothalamus. Tanycytes are provided with a single basal process that ramifies into the underlying neuropil, contacting neurons and glial cells and/or ending on capillaries (Rodríguez et al., 2005). Osmophilic inclusions and numerous polymorphic and round vesicles have been described by transmission electron microscopy within the endfeet of tanycytes, suggesting a possible secretory role for them (Monroe and Paull, 1974). Thus, the CNTF upregulation seen in HFD mouse tanycytes and, notably, their stained processes, may be morphological features reflecting an increased synthesis and secretion of CNTF, which in turn diffuses by volume transmission in the neuropil and reaches distant neurons. However, the considerable mismatch between the putative site of CNTF production and secretion (tanycytes) and its putative central targets (likely the second-order neurons of the energy balance circuits) suggests the interesting possibility that CNTF may be a circulating factor capable to affect the hypothalamic neuronal circuits of the energy balance in HFD conditions.

## Author contributions

Ilenia Severi performance of experiments, data analysis and interpretation, manuscript writing. Jessica Perugini, Eleonora Mondini, Arianna Smorlesi, and Andrea Frontini performance of experiments and data analysis and interpretation. Saverio Cinti critical revision of the manuscript, financial support. Antonio Giordano conception and design, financial support, data analysis and interpretation, manuscript writing, final approval of the manuscript.

### Conflict of interest statement

The authors declare that the research was conducted in the absence of any commercial or financial relationships that could be construed as a potential conflict of interest.
